# Environmental Practices in Firms Located in Underpopulated Rural Areas in Spain: The Case of the Province of Teruel

**DOI:** 10.3390/ijerph17238993

**Published:** 2020-12-02

**Authors:** Luisa Esteban-Salvador, Ana Felicitas Gargallo-Castel, Javier Pérez-Sanz

**Affiliations:** 1 Department of Accounting and Finance, University of Zaragoza, 44003 Teruel, Spain; luisaes@unizar.es; 2 Department of Business Management and Organisation, University of Zaragoza, 44003 Teruel, Spain; fjperez@unizar.es

**Keywords:** rural, environmental practice, Spain, underpopulated areas

## Abstract

This study aims to develop a better understanding of what drives small and medium-sized enterprises (SMEs) to engage in environmental practices in isolated rural areas. Despite a growing literature on environmental behavior in different contexts, the green activities of SMEs in rural areas remains underexposed. This neglect is remarkable, and deserves attention given the serious depopulation problems they have to face, and the economic and social challenges that lie ahead. Using unique data from 141 SMEs in one of the most sparsely populated regions in the European Union, we study the influence of territorial relations on firms’ environmental conduct. Our results suggest that different territorial factors have some impact on the adoption of environmental practices. We report evidence indicative of the role of these factors in shaping environmental decisions. Finally, we offer suggestions for future research that could further develop our understanding of environmental management decisions in rural and underpopulated areas.

## 1. Introduction

The more remote rural and sparsely populated areas in the countries of the European Union have in common weaker economic growth than those close to urban areas [1]. In Spain, the debate on the shortcomings of depopulated rural areas with respect to urban ones has gained significant inertia. The term “emptied Spain” is becoming more and more frequent in the media. Therefore, it is necessary to define a sustainable plan strategy for rural Spain that promotes economic and social development.

According to the Organisation for Economic Co-Operation and Development (OECD) [2], changes affecting rural areas have several key impacts. Firstly, rural regions are faced with continued migration to urban areas and an ageing population, which poses serious economic and social challenges. Secondly, these demographic profiles erode the population needed to preserve public services and develop economic activities, creating a vicious circle that is difficult to break. Teruel is one of the Spanish provinces which is most affected by such depopulation. Located in the northeastern autonomous community of Aragon, Teruel has a surface area of 14,809 km^2^ and a total population of 134,137 inhabitants, of which 24.09% are aged 65 years or older [3]. The province also has a high rate of over-ageing, with 24.44% of the elderly population being 85 years old or more. Problems of depopulation and lack of services are not new. In 1999, a spontaneous popular movement for the development of the province emerged in Teruel, supported by all the economic, social and political organizations of the area. It expresses a desire for knowledge and recognition.

The unique characteristics of Teruel makes it a relevant case for the proposed analysis. With the pandemic caused by the coronavirus, the province’s lack of health facilities and infrastructure, as well as the shortage of services or the scarcity of internet access in many of the small towns have exacerbated the situation. Moreover, in this context, governments have a golden opportunity to ensure a sustainable recovery from the economic impact of the Covid-19 crisis by focusing on green recovery as a win-win strategy. Many governments have included “green” recovery measures in their crisis recovery plans (the OECD’s preliminary estimates indicate some US $312 billion of funding), such as through tax relief or grants for green transportation, circular economy and clean energy research [4]. In addition, the European Union has approved a budget for the 2021–2027 period focused on investment in the green and digital transition [5] in order to aid the reconstruction of the member countries after the pandemic produced by Covid-19.

Most studies of environmental practices have relied on large companies. This gap has spurred an increase in small and medium-sized enterprises (SMEs) in recent years [6,7,8]. Although the footprint of individual SMEs is low, since they make up the largest percentage of companies, their aggregate impact can have a substantial environmental impact, and even exceed that of large companies [8]. Nevertheless, it is worth noting that despite the growing literature on environmental behavior in different contexts, research on rural SME environmental conduct is practically non-existent. This study helps to fill this gap. The main objective of this paper is to develop a better understanding of the factors that influence the engagement of SMEs in environmental practices in rural territories because according to the OECD [9] “SMEs are important for green growth as key drivers of eco-innovation and key players in emerging green industries”.

The present study also contributes to the understanding of the environmental behavior of small and medium enterprises in a rural area classified as sparsely populated by the European Union, and where many small towns have disappeared or are in danger of disappearing. Researchers have also neglected this area of study, and therefore it has been scarcely analyzed. For this reason, the study is essential within the framework of the literature on depopulated areas and environmental practices. The research provides evidence of the positive effects of the territorial linkage and the responsibility of stakeholders in predicting the environmental performance of rural SMEs. Moreover, this paper adds to theoretical perspectives on environmental practices in sparsely populated areas in the European Union.

In addition, this paper contributes to answering the research question on the determinants of environmental performance of enterprises, and offers a distinctive value due to its focus on SMEs in a very specific context. It goes into detail on the relationship between the introduction of environmental practices in rural SMEs and the links between these enterprises and the territory.

The paper is organized as follows. In the next section, we present a review of the theoretical framework linked to environmental practices in rural territories. Later, we establish the hypothesis and define the methodology to be followed, which is based on logistic regression. Then we present the results, and finally, the conclusions in which we include some of the study’s limitations and ideas for future research.

## 2. Theoretical background

### 2.1. Rural Areas

**Figure 1:**
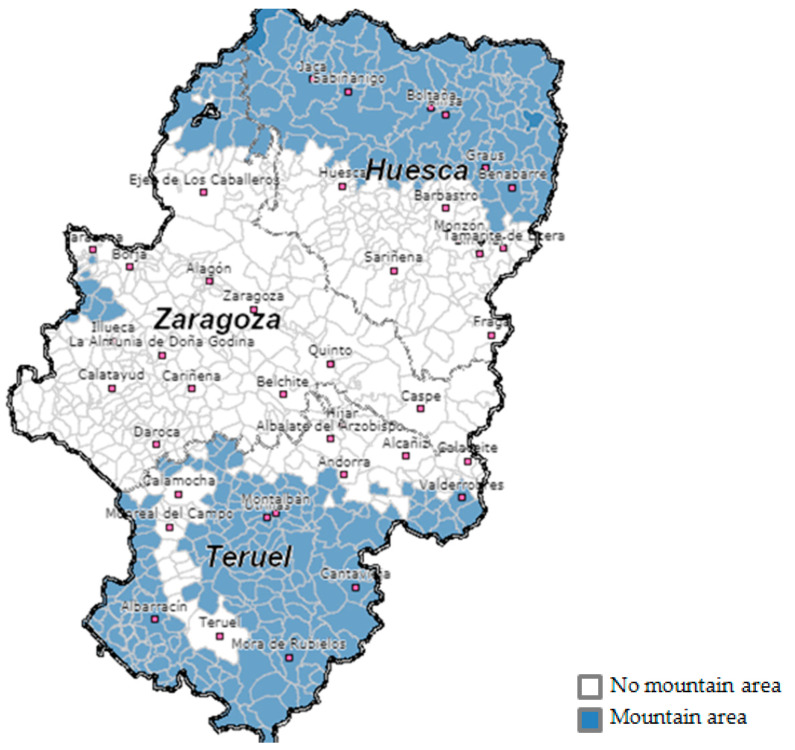
Map of mountain areas in Aragon. Source: Government of Aragon [10].

Sustainable development is interdependent with the development of the rural world. Rural areas represent approximately three-quarters of the land and account for a quarter of the population of OECD countries [2]. They also represent a strategic asset for economic growth, human development and environmental balance. At a time of increasing globalization, the processes of expansion of urban areas and the ageing of rural populations have led to significant transformations. One of the disadvantages of living in remote rural areas is access to healthcare “while people living in rural areas are more likely to be deterred from seeking health care services as a result of travelling long distances (medical services tend to be concentrated in towns and cities), and the length of waiting lists reflects the supply of and the demand for services (which may vary according to the treatment, therapy or intervention required)” [1]. As an exception, in rural areas with relatively high levels of accessibility and those characterized by climatic advantages or other amenities (such as in France, England and Italy), “urban to rural” migration is occurring [2]. However, in the case of Teruel, 178 of the total number of municipalities in the province are classified as mountainous areas and the other 58 as disadvantaged areas according to the Department of Agriculture, Livestock and the Environment of the Government of Aragon [10] (Figure 1).

**Figure 2:**
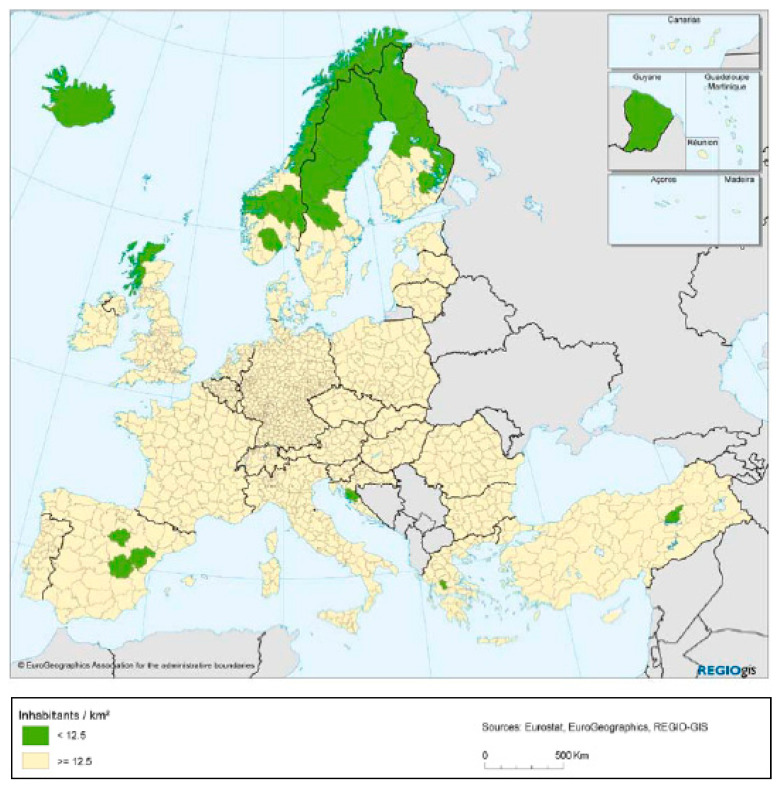
Sparsely populated regions. Source: Eurostat [12].

Teruel is one of the “predominantly rural” regions of Europe, according to the urban–rural typology presented by the OCDE [11]. In particular, the province has no urban centers with more than 200,000 inhabitants, and the proportion of the population living in local rural units with a population density below 150 inhabitants/km^2^ is more than 50%. Moreover, Teruel is one of the most sparsely populated regions in the European Union [12] (Figure 2), with less than 12.5 inhabitants/km^2^.

In this context, as a result of the process of depopulation in the province, the lack of public investment and a feeling of marginalization and abandonment, in 1999 a citizen’s coordinator emerged to support to the development of the region (“Teruel does exist!”). This is an example of social capital that has no equivalent in the national territory [13].

### 2.2. Territory and Environmental Practices

Some studies suggest social, regulatory, and economic pressures shape a license to operate which guides firms’ environmental practices. This “social license” has been defined as “the demands on and expectations for a business enterprise that emerge from neighborhoods, environmental groups, community members, and other elements of the surrounding civil society” [14]. The social license to operate (SLO) theory is increasingly being used throughout the world to describe a specific aspect of company-community relations. In particular, the SLO concept has proven useful for describing the relationship between industry and local communities [15].

Some authors [14,16] investigated why corporate environmental performance differs among firms. This research question led to the emergence of the generalization of social license as part of a broader explanatory framework to explain why certain companies go beyond merely complying with environmental regulations. The results of in-depth research on environmental performance suggest that firms, which are locally highly visible rely not only on economic and legal requirements but also on social license and local community demands. Some authors explore the usefulness of social license in the implementation of sustainable development strategies in delimited territories, highlighting the principle of “subsidiarity” where development decisions should be taken closer to the local community [17]. The institutional strategy “Rural Development Program of Aragon 2014–2020” [18], where the rural areas of the study are located, is based on improving the situation of the population in rural areas through the development of services and the generation of rural employment. It represents a commitment to the sustainable management of natural resources and initiatives aimed at balanced territorial development and climate concerns.

This approach fits with the enterprises located in rural areas, in their commitment to the development of social responsibility vectors, and specifically with the environmental performance as a guarantee of commitment in the preservation of the territory and the improvement of the quality of life for rural inhabitants [19]. Taking advantage of business opportunities based on activities such as respectful and responsible tourism in small rural villages can have a positive effect on sustainable rural development [20]. In this regard, business strategies based on a commitment to environmental values promote models of sustainable and responsible management of hospitality establishments in the rural environment, with positive effects on the competitive position of the sector. This allows them to overcome the negative impacts of rural tourism and enable environmental sustainability [21,22].

The influence of stakeholders on the adoption of environmental practices has been well established in literature [23,24]. Over recent decades, firms have become more aware of the importance of implementing environmental strategies because of the growing stakeholder pressure [25,26]. Other authors [27] analyze the degree of application of social responsibility in the performance of rural SMEs. They highlight a greater involvement in environmental sustainability, motivated by energy-saving, pressure from local stakeholders and the adoption of their customers’ lifestyle values. The influence of environmental variables has been analyzed in the management of small rural companies by observing a segmentation of business behaviors based on savings and efficient resources management, as an opportunity to improve competitiveness and as a tool to obtain short term profit [28]. In the study carried out by GHK Consulting Ltd. for the Department for Environment Food and Rural Affairs (DEFRA) in 2011 titled “microbusinesses and environmental regulation” [29], some of the incentives for micro-enterprises to carry out environmental practices were found to be the improvement of local image or an increase of customers. More recent research concludes that the implementation of sustainability practices by small rural tourism businesses is determined by environmental pressures, mainly from their clients. This issue is associated with the concept of social license, which stimulates the establishment of control mechanisms to evaluate the amount of social responsibility exerted and strengthens positive attitudes towards sustainable initiatives [30]. In addition, some research found that consumers that are environmentally concerned “created a common sense of responsibility and commitment that is expressed through their ethical organic food consumption and production behaviors, global environmental sensitivity, and by their actions within their communities” [31].

In Spain, article 1.c of Law 45/2007 of 13 December on the Sustainable Development of the Rural Environment [32] sets among its objectives the conservation and recovery of the heritage and natural and cultural rural resources through public and private actions that allow their use while being compatible with sustainable development. Likewise, article 1.e aims to achieve a high level of environmental quality in rural areas, preventing the deterioration of the natural heritage, landscape and biodiversity, or facilitating their recovery. This is to be achieved through the integrated management of territory usage for different activities, improved planning and management of natural resources and reduction of pollution in rural areas. In addition to complying with legal regulations, Spanish SMEs can also address the needs of the local social and natural environments. This rural setting in the province of Teruel could present points of similarity with those shown in other studies [14], where authors observe that firms do not perceive their social obligations only by contemplating legal criteria, but by including the conditions and demands required by different social agents, which often exceed those stipulated legally.

From the corporate social performance perspective [33,34], companies are social institutions that must operate responsibly, within the expectations and control of the society in which are subject to so-called “social contracts” [35], and seek transparency and durability in this relationship. According to the social contract idea, businesses have a moral obligation to act in a socially responsible manner, which is the basis of an intangible social agreement with society [36]. In rural settings, there is a real need to collaborate between different actors because “sparsely populated areas with large distances between communities impose high costs of running a business; for example, costs of transport are higher than in urban areas” [37].

Moreover, enterprises could see that the application of environmental practices in territories where there is a close relationship with the inhabitants could improve its public image with customers, suppliers, public administrations, workers, other companies, and the general public. In environments in which the company is familiar with the traditions, culture, problems, hobbies or concerns of its stakeholders, socially responsible practices could lead to an improvement of its image. The promotion of culture, health, and nutrition are listed as rural development strategies [38]. Some evaluated firms indicated that one way to integrate into society is through their involvement in community events and fairs such as the promotion of local activities, sports, and traditions, and support of local communitarian facilities, among the strategies of culture and identity preservation [38]. Stakeholders can exert pressure, but they can also provide the company with useful information. Moreover, customer derived information, suppliers and other stakeholders can be helpful to offer products and services adapted to their needs and to better understand their preferences in terms of environmental protection. Therefore, communication with stakeholders may increase the propensity to adopt environmental actions. Principle 10 of Agenda 21 of United Nations Conference on Environment and Development [39] specifies that all citizens must have the channels to know and be able to deal with environmental issues in their communities and participate in decision-making processes according to their needs. Some authors point out the importance of cooperation as a key to success in the implementation of corporate social responsibility strategies, “managers should facilitate communication with local communities and raise awareness of the company’s intentions among stakeholders and therefore develop a deeper understanding between them and the company” [40]. Other studies [41] highlight the importance of interacting and establishing communication channels with different stakeholders for corporate social responsibility because this helps to identify their specific demands [42,43,44]. A desire for collaboration and cooperation between different actors is patent given that, “in rural areas, there is a real need to make changes and/or deliver a service or product that is currently unavailable” [37]. As the interactions with stakeholders increase, their contribution to environmental strategy is enriched and is often accompanied by more innovative solutions that enjoy greater social legitimacy [45]. Grounded in legitimacy theory, organizations should meet stakeholders’ expectations, respecting their social norms and values [46,47]. Legitimacy is defined as a generalized perception that the actions of an entity are congruent with the values of the larger social system of which the organization is a part [48]. Therefore, legitimacy reflects the predominant norms and values in the local community. In particular, in the context of sparsely populated rural areas, legitimacy may have a pivotal relevance with an existing social contract which involves entities that operate in this territory and the small social community that supports them.

In this regard, some authors found support for legitimacy theory as an explanatory factor for environmental disclosures [36,49,50].

Corporate social responsibility and environmental management provide practical resources to the organizations concerned about social and environmental responsibility in the context of sustainable development [51,52]. Moreover, companies that already develop other socially responsible behaviors in the local community are expected to be more sensitive to the development of practices aimed at environmental protection. Strong collaboration in local social activities reflects an interest in the territory and its people.

The notion of territory denotes a spatial and geographical area subject to management and transformation measures in terms of the living environment [53]. This notion territory image is similar to the term “landscape”, used to designate parts of a territory as perceived by their inhabitants [54]. Greater attachment to the territory and the feeling of belonging, typical of rural areas with reduced dimensions and closely related society, can be transmitted by the local community to the companies and favor their active participation through the development of environmentally responsible practices to preserve the territory, a sign of collective identity. There is some support for the claim that a sense of belonging and community ties are more substantial in rural areas [55]. Therefore, according to SLO, firms in small rural areas could be more sensitive to rural community groups and community demands to protect the natural environment [56].

Therefore, Hypothesis 1 was formulated:

**Hypothesis** **1.** *There is a positive relationship between the introduction of environmental practices in rural SMEs and the link of these enterprises with the territory.*

## 3. Methodology

The objective of this study is to better understand the influence of several strategic decisions in the adoption of environmental actions in a representative sample of Spanish SMEs of the province of Teruel (Spain). We intend to find out if the care and protection of the environment are related to a series of aspects linked to the geographical area in which the activity takes place, characterized by being highly depopulated, and with close connections between citizens and companies.

To carry out the study, we designed a survey that was conducted in 2018. In a previous study, we analyzed the entire population of all non-financial Spanish SMEs in the province of Teruel taken from the SABI (System of Analysis of Iberian Balances) database. We received a response from 511 companies. In 2018 we sent a new questionnaire to all the companies that responded to the previous one, this time focused on environmental issues, and we received 141 responses, representing a rate of 27.59%.

The questionnaire was prepared and sent by electronic mail to all the potential respondents. When the electronic questionnaires were returned or not answered, we interviewed the possible respondent by phone. In the introduction to the interview, we stated that the answers were going to be treated anonymously, and we would maintain confidentiality in the management of the data. This is intended to reduce reluctance to answer for fear that the information may be misused [57].

The general approach taken here is to arrive at a prediction model for companies environmental practices. The values of dependent variable of the model is 1: the company carries out substantial environmental practices or 0: the company carries out no or minimal environmental practices. The variable is predicted by analyzing a series of other variables related to the decisions the company takes with respect to the local territory. These predictive variables are to be collected through a questionnaire. To find out to what extent SMEs carry out environmental activities, we ask the following question: does the firm carry out actions for the care of the environment? The response options followed a Likert scale from 1 to 5, with the possibility of leaving the question blank or unanswered.

We also asked how many different practices the firms carried out. After analyzing the responses, we observed that when the answer was 1 or 2, the environmental practices were minimal. In contrast, when the answer was between 3 and 5, the firms did indeed carry out relevant environmental practices. For this reason, we coded 0 when the enterprise did not implement environmental practices or when they were minimal (answers 1 or 2) and 1 when the firm performed higher levels of environmental practices (answers 3, 4 or 5).

To test our hypotheses, we developed a questionnaire with the items set out in Table 1. In the prediction model, we used all the items except those that could present multicollinearity problems after the analysis had been performed. We used responses to the questionnaire to construct the variables. We used a five-scale instrument to measure the agreement or disagreement with the questions. Moreover, we obtained information from secondary sources for the area in square kilometers of each location.

Table 1 shows the list of variables.

In this study, we try to build up a model to predict the implementation of environmental practices using the different items in the survey and a control variable. The dependent variable is binary; zero or basic environmental practices and high environmental practices. Since we are interested in the odds of high environmental practices, the reference category is zero or basic environmental practices. Therefore, high environmental practices are coded as one and zero or basic environmental practices as zero. Based on the sample size, the research questions, and our binary dependent variable, we have chosen to use a logistic regression model to test our hypotheses. Before building the model, we have verified that the number of variables is within those recommended for the sample size in order to improve the precision of the model [58,59]. We have performed the multicollinearity diagnoses with all the predictor variables, using the variance inflation factor (FIV), and no coefficient had a value greater than 10, nor were the tolerance coefficients less than 0.10. However, the condition index, with all the items related to the questions, was high, with a value of 25. With this in mind, we have adjusted the data and eliminated one of the questions (Q8) that could present multicollinearity problems, thereby lowering this index to 18.57.

The general form of a logistic regression equation from which *P*(*Y*) is the probability of *Y* occurring, *e* is the base of natural logarithms, and β are the coefficients (Equation (1)):

*P(Y)* = 1/1 + *e*^−(*β*^^_0_^^+*β*^^_1*i*_^*^x^*^_1*i*_^^+*β*^^_2*i*_^*^x^*^_2*i*_^^+*…*+*β*^*^_ni_^^x^^_ni_^*^)^ (1)

Therefore, for a given enterprise, *P*(*Y*) will be a value between zero and one, where zero is no chance that the firm carries out important environmental practices, and one is the certainty that the firm develops high environmental practices. The predictor variables are represented by the answers to the questions that were asked to interviewees (Table 1). As a control variable, we take the surface are of the municipality in which the companies are installed, measured in square kilometers. We developed the logistic regression equation with the predictor and control variable as (Equation (2)):

*ln(odds high environmental practices)* = *β*0 + *β*1 *Q*1 + *β*2 *Q*2 + *β*3*Q*3 + *β*4*Q*4 + *β*5*Q*5(1) + *β*6*Q*6 + *β*7*Q*7 + *β*9*Q*9 + *β*10 *Surface* (km^2^) + *ε* (2)

A positive value of *R*^2^ indicates that as the predictor variable increases, so does the likelihood of the firm developing high environmental practices. A negative value implies that as the predictor variable increases, the probability of the outcome decreases. If a variable has a small value of *R*^2^, then it contributes only a small amount of the model. The odds ratio (*Exp (B)*) of an event occurring are defined as the probability of an event occurring divided by the likelihood of that event not occurring (Equation (3)).

*Odds = P (event)/P (no event)* *P(event Y) = 1/1+e^−(β^*^_0_^*^+β^*^_1*i*_^*^x^*^_1*i*_^*^+β^*^_2*i*_^*^x^*^_2*i*_^*^+…+β^^_ni_^^x^^_ni_^* (3)

## 4. Results

Table 2 shows the descriptive statistics of the variables included in the questionnaire before creating the prediction model. As can be seen, more enterprises carry out significant environmental practices than those that do not or those that contribute to improving the environment do so in a very basic way. In both cases, the firms that claim to carry out effective environmental practices, as well as those that either do not carry out or the actions they take are minimal, what they value most is that the enterprise’s products or services meets the needs of individuals or businesses in the area. However, this variable is not included in the model to avoid multicollinearity problems.

As shown in Table 2, the firms that develop low environmental practices have achieved a lower score than the firms that do perform these practices, in all the questions of the questionnaire. The results show that firms with superior environmental practices also stand out because of the importance that their desire to contribute to the development of the city or region had in choosing their location. These enterprises obtain a score of 4.16 compared to 3.53 points of the firms that either do not carry out environmental practices or these are very scarce. The score for the question “in the promotion of the products, some kind of allusion to the town, region, natural area, etc., is used” obtains an average evaluation of 2.97 in enterprises that are less friendly to the environment and 3.20 for the friendliest.

Regarding the promotion of the products, indirectly promoting the area (for example, publicising the gastronomy, the customs, the towns, other products in the area, etc.) the valuation of the less environmentally sensitive SMEs is below the average (2.40). Although the score of the firms most aware of the environment is above the average, it is not very high (2.64).

Concerning suppliers, there is a general preference for selecting suppliers from the area, but this preference is greater in companies that are more sensitive to the environment (3.72) compared to the rest (3.03).

In general, the responses suggest that firms gather useful information from the environment (suppliers, customers, workers, society, …) in order to improve their products or services, and that this variable reaches a very high valuation in firms that implement environmental practices, with a valuation of 4.31 compared to 3.48 of the less sensitive ones.

The use of the Internet does not seem to have influenced the increase in purchases outside the area, in fact, SMEs that do not carry out environmental practices or these are low, obtain a score below the average (2.48). All firms indicate that the Internet makes it easy to maintain a location in rural areas (because it has allowed reducing travel, reaching more customers, access to suppliers, advertising online, …), obtaining a valuation of 3.55 in the case of firms not friendly with the environment and 3.77 in the case of those sensitised with the environment.

The high score given to the question “the firm contributes with its products or services to meet the needs of individuals or companies in the area” is striking. All SMEs agree with this contribution, with a valuation of 4.39 points in the case of non-environmentally friendly enterprises, compared to 4.57 in those that are environmentally friendly. In both cases, SMEs consider that their role is important in local clients, both for individuals and for other firms.

Firms that are less sensitive to the environment are not sensitive to collaboration in local cultural and sports activities, etc., for example, sponsors sports teams, contests, awards …, give a score below the average (2.27). This is not the case with environmentally friendly ones, where they value this aspect at 3.02. This means that firms less committed to respecting the environment are not involved to their closest stakeholders either.

The main results of the model of logistic regression in our research are presented in Table 3. The full model significantly predicted high environmental practices (omnibus chi-square = 34.01, df = 9, *p* < 0.00). The model accounted for between 27 per cent and 42 per cent of the variance in high environmental practices, with 86.9 per cent of the increased environmental practices successfully predicted. Table 3 gives coefficients, the Wald statistic and associated degrees of freedom, and probability values for each of the predictor variables. The model shows that questions Q5, Q9 and Surface (km^2^) reliably predicted high environmental practices. The value of the coefficients of the Q5 (the company obtains useful information from the environment (suppliers, customers, workers, society, …) to improve its products or services), and of the Q9 (the firm collaborates in local cultural and sports activities, etc., for example, sponsors sports teams, contests, awards …) reveal that an increase in the value of this predictors is associated with an increase in the odds of high environmental practices. On the contrary, the surface (km^2^) is negatively associated with high environmental practices.

Furthermore, item Q5 suggests that when a firm obtains useful information from the environment to improve its products or services, the likelihood that it implements high environmental practices is 1.975 times greater than whether the firm did not. Therefore, when the firm collaborates in local cultural and sports activities, etc., for example, sponsors sports teams, contests, and awards, it is more likely that the enterprise develops high environmental practices. Further, the increase in square kilometres of the area in which the firm is located predicts low or no environmental practices.

## 5. Discussion

The research provides several important contributions. The study adds evidence to the positive effects of the territorial link and the accountability to the stakeholders in the prediction of environment-friendly businesses. Furthermore, when enterprises are located in small towns, their activity to take care of the environment is more significant. Perhaps this is so because in rural settings social networks are deeper than in urban settings [37,60]. Therefore, the study contributes to complementary theoretical perspectives on environmental practices on sparsely populated regions in the European Union.

There are several theoretical and practical implications for SMEs, researchers and politicians. From a theoretical point of view, the results confirm the adequacy of the legitimacy theory and the social contract framework to study the relationship between the environmental behavior of rural SMEs and the link of these enterprises with the territory. Practical implications also emerge. It is important to promote the exchange and gathering of useful information from internal and external stakeholders in order to encourage the development of environmental practices. Public policy initiatives to promote such contexts are therefore welcome. Additionally, efforts are also needed to improve the territorial identity strategy to enhance the environmental performance linked to the promotion of rural products and services as a differentiated element.

Furthermore, the present study has limitations that can be resolved in future studies, such as identifying environmental practices with different sectors of the economy, or how the coronavirus pandemic has affected companies located in these populations and their environmental practices as well as public health.

Future studies could replicate this research in other regions or countries. Moreover, it would be interesting to extend the present study to include more territorial elements and different methodologies, including qualitative techniques, as well as a longitudinal analysis to examine the evolution of results over time.

## 6. Conclusions

Policymakers and development specialists have recognized social and environmental responsibility as a feasible driver for rural development [38]. This study extends the work on environmental practices in SMEs located in the province of Teruel in Spain, one of the most sparsely populated regions in the European Union with less than 12.5 inhabitants/km^2^. The research drew on a sample of businesses in this province to advance our understanding of the characteristics of firms that declare their intent to carry out environmental practices.

To find the questions which best discriminate between firms that claim to carry out critical environmental practices versus those that do not take care of the environment or do so with little intensity, we have conducted a binomial logistic regression. In the model, we found three statistically significant questions contributing to the prediction of businesses carrying out environmental practices.

Based on these results, we can conclude that the probability that a firm undertakes environmental practices is higher in firms that obtain useful information from the environment (suppliers, customers, workers, society, etc.) to improve its products or services, collaborates in cultural and sports activities, etc., of the area, for example, sponsors sports teams, contests, or awards that are located in smaller municipalities. This may be related to two factors: a greater attachment to the territory and the feeling of belonging, and the greater strength of the “social license to operate” requirement demanded by the local community, where the group is less numerous, but more closely related. This is in line with other studies that found a relationship between social legitimacy and the environmental management practices of companies [61]. This conclusion also has similarities with the results obtained in other studies that reflect the pressure from stakeholders for appropriate environmental protection in business practices [62,63,64]. Socially responsible behavior reflected in strong collaboration in local activities is also essential. Finally, clear communication with stakeholders allows the firm to be sensitive to their needs, which increases the propensity to adopt environmental actions and allows enterprises to take advantage of collective learning and seek new ways to solve environmental problems [65]. The close relationship of a firm with its stakeholders could generate feedback in which the information about their needs drives the improvement of their products and services. In fact, “in rural areas, businesses need to remain flexible and open to delivering a range of needed products and services” [37]. In addition, this interconnection could lead to a local social commitment through financial collaboration in activities in the area such as the sponsorship of local sports teams, or the launch of contests, prizes or the carrying out of cultural activities.

Digitalization offers new possibilities to SMEs in rural areas [66]. Nevertheless, some questions related to the Internet have not been statistically significant to predict environmental practices, for example, that the use of the Internet contributes to the increase in the percentage of purchases outside the area, or that the Internet makes it easier to maintain the location in the rural environment because it has allowed reduced travel, more customers reached, better access to suppliers or online advertising. Perhaps it is necessary to bear in mind that many of these villages do not have access to the Internet. Digital investment in rural areas is, together with the green transition, one of the bets of the European Union for the next seven years [67]. For them, it is hoped that with the application of European funds for the recovery from the pandemic, the digital divide in the small towns of the province will decrease. Although several actions have been developed in recent years to fight against rural depopulation and the consequent loss of education and healthcare services which rural recession involves [68], more investments are needed.

On the other hand, the study did not find that the promotion of products with differentiated brands in rural areas was significant, possibly due to a lack of territorial identity strategy as an element of differentiated quality in the products and services offered [69] that would allow a greater social benefit to the rural space [70,71], such as the labelling of organic agricultural products [72].

## Figures and Tables

**Table 1:** Question items.

Dependent Variable:	1 = The Firm Carries out High Environmental Practices 0 = The Firm Does Not Carry out Environmental Practices or That They are Minimal
Predictor variables:	
Q1	When choosing the location, the desire to contribute to the development of the town or the region was important.
Q2	In the promotion of your products, some kind of allusion to the town, region, natural area, etc. is used.
Q3	In the promotion of products, an indirect promotion of the area (for example, mentioning the gastronomy, the customs, the towns, other products in the area, etc.).
Q4	The firm selects its suppliers and puts those from the area first.
Q5	The firm obtains useful information from its context (suppliers, customers, workers, society, …) to improve its products or services.
Q6	With the use of the Internet, the percentage of purchases outside the area has increased.
Q7	The Internet makes it easy to maintain a location in rural areas (because it allows reduced travel, more customers reached, better access to suppliers, online advertising, …)
Q8	The firm contributes with its products or services to meet the needs of individuals or companies of the area.
Q9	The firm collaborates in cultural and sports activities, etc. of the area, for example, sponsors sports teams, contests, awards.
Control variable:	Surface area (km^2^)

Source: Own elaboration. Q*i* = Question *i*, where *i* = 1–9.

**Table 2:** Statistics of the variables.

Environmental Practices			Q1	Q2	Q3	Q4	Q5	Q6	Q7	Q8	Q9	Surface (km^2^)
Null or scarce	N	Valid	32	29	25	33	33	33	33	33	33	33
		Missing	1	4	8	0	0	0	0	0	0	0
	Mean		3.53	2.97	2.40	3.03	3.48	2.48	3.55	4.39	2.27	304.31
	Median		3.00	3.00	2.00	3.00	4.00	2.00	4.00	5.00	1.00	440.41
	Mode		5	1	1	1 ^a^	5	1	5	5	1	440.41
	S.D.		1.344	1.614	1.607	1.741	1.482	1.482	1.563	0.933	1.663	162.86
	Minimum		1	1	1	1	1	1	1	1	1	26.76
	Maximum		5	5	5	5	5	5	5	5	5	472.12
	Sum		113	86	60	100	115	82	117	145	75	10042.28
High	N	Valid	107	90	84	107	108	108	107	108	108	108
		Missing	1	18	24	1	0	0	1	0	0	0
	Mean		4.16	3.20	2.64	3.72	4.31	2.78	3.77	4.57	3.02	248.95
	Median		5.00	4.00	3.00	4.00	4.50	3.00	4.00	5.00	3.00	142.80
	Mode		5	5	1	5	5	1	5	5	1	440.41
	S.D.		1.175	1.677	1.618	1.510	0.837	1.555	1.470	0.823	1.547	176.00
	Minimum		1	1	1	1	1	1	1	1	1	20.97
	Maximum		5	5	5	5	5	5	5	5	5	472.12
	Sum		445	288	222	398	465	300	403	494	326	26,886.74

^a^ Multiple modes exist. The smallest value is shown. (S.D.: standard deviation). Source: own elaboration.

**Table 3:** Logistic regression of predictors of high environmental practices.

Dependent Variable:	Model				
	Environmental Practices				
	B	S.D.	Wald	df	Exp (B)
Q1: When choosing the location, the desire to contribute to the development of the town or the region was important.	0.322	0.203	2.532	1	1.380
Q2: In the promotion of the products, some kind of allusion to the town, region, natural area, etc. is used.	0.202	0.262	0.594	1	1.224
Q3: In the promotion of the products, an indirect promotion of the area (for example, giving know the gastronomy, the customs, the towns, other products in the area, etc.).	−0.495 *	0.282	3.077	1	0.609
Q4: The firm selects its suppliers, putting them first from the area.	0.213	0.214	0.997	1	1.238
Q5: The firm obtains useful information from the environment (suppliers, customers, workers, society, etc.) to improve its products or services.	0.681 ***	0.257	7.029	1	1.975
Q6: With the use of the Internet, the percentage of purchases outside the area has increased.	0.295	0.227	1.691	1	1.343
Q7: Internet makes it easy to maintain a location in rural areas (because it has allowed reducing travel, reaching more customers, access to suppliers, advertising online, etc.).	−0.058	0.203	0.080	1	0.944
Q9: The firm collaborates in local cultural and sports activities, etc., for example, sponsors sports teams, contests, awards, etc.	0.472 **	0.216	4.770	1	1.603
Surface (km^2^)	−0.005 ***	0.002	7.037	1	0.995
Constant	−3.021 **	1.455	4.310	1	0.049
Cox and Snell R Square	0.27				
Nagelkerke R Square	0.42				
Chi-square	34.01 (df = 9, *p* < 0.00)				
Classification % correct	86.9				

Note: B: coefficients; S.D.: standard deviation; Wald: Wald statistic; df: degrees of freedom; Exp (B): odds ratio The levels of significance are * *p* < 0.1; ** *p* < 0.05; *** *p* < 0.001. Source: own elaboration.
